# Peripheral Single‐Cell Immune Characteristics Contribute to the Diagnosis of Alzheimer's Disease and Dementia With Lewy Bodies

**DOI:** 10.1111/cns.70204

**Published:** 2025-01-03

**Authors:** Conglong Qiu, Danhua Zhang, Majie Wang, Xi Mei, Wei Chen, Haihang Yu, Weiwei Yin, Guoping Peng, Shaohua Hu

**Affiliations:** ^1^ Department of Psychiatry The First Affiliated Hospital, Zhejiang University School of Medicine Hangzhou China; ^2^ Department of Psychiatry Affiliated Kangning Hospital of Ningbo University Ningbo Zhejiang China; ^3^ Department of Psychiatry Ningbo Kangning Hospital Ningbo Zhejiang China; ^4^ Key Laboratory for Biomedical Engineering of the Ministry of Education, College of Biomedical Engineering and Instrument Science Zhejiang University Hangzhou China; ^5^ Department of Cell Biology and Cardiology Second Affiliated Hospital, Zhejiang University School of Medicine Hangzhou China; ^6^ Liangzhu Laboratory Zhejiang University Hangzhou China; ^7^ Zhejiang Provincial Key Laboratory of Cardio‐Cerebral Vascular Detection Technology and Medicinal Effectiveness Appraisal, College of Biomedical Engineering and Instrument of Science Zhejiang University Hangzhou China; ^8^ Department of Neurology The First Affiliated Hospital, Zhejiang University School of Medicine Hangzhou Zhejiang China; ^9^ Nanhu Brain‐Computer Interface Institute Hangzhou China; ^10^ The Zhejiang Key Laboratory of Precision Psychiatry Hangzhou China; ^11^ MOE Frontier Science Center for Brain Science and Brain‐Machine Integration Zhejiang University School of Medicine Hangzhou China; ^12^ Brain Research Institute of Zhejiang University Hangzhou China; ^13^ Department of Psychology and Behavioral Sciences Zhejiang University Hangzhou China; ^14^ Zhejiang Engineering Center for Mathematical Mental Health Hangzhou China

**Keywords:** Alzheimer's disease, dementia with Lewy bodies, single‐cell analysis

## Abstract

**Objective:**

Alzheimer's disease (AD) and dementia with Lewy bodies (DLB) are common neurodegenerative diseases with distinct but overlapping pathogenic mechanisms. The clinical similarities between these diseases often result in high misdiagnosis rates, leading to serious consequences. Peripheral blood mononuclear cells (PBMCs) are easy to collect and can accurately reflect the immune characteristics of both DLB and AD.

**Methods:**

We utilized time‐of‐flight mass cytometry (CyTOF) with single‐cell resolution to quantitatively analyze peripheral PBMCs, identifying 1228 immune characteristics. Based on the top‐selected immune features, we constructed immunological elastic net (iEN) models.

**Results:**

These models demonstrated high diagnostic efficacy in distinguishing diseased samples from healthy donors as well as distinguishing AD and DLB cases. The selected features reveal that the primary peripheral immune characteristic of AD is a decrease in total T cells, while DLB is characterized by low expression of I‐kappa‐B‐alpha (IKBα) in the classical monocyte subset.

**Conclusions:**

These findings suggest that peripheral immune characteristics could serve as potential biomarkers, facilitating the diagnosis of neurodegenerative diseases.

## Graphical abstract

By observing the single‐molecule immune profiles of peripheral blood PBMC cells in AD, DLB, and healthy populations, we have discovered that the cyTOF technology can efficiently diagnose these two types of diseases and may attempt to discover potential molecular immune differences.

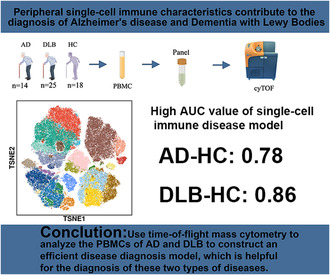

## 1 Introduction

Neurodegenerative diseases significantly impact human health. Among these, Alzheimer's disease (AD) and dementia with Lewy bodies (DLB) are the most prevalent, ranking first and second in incidence among all types of dementia [1]. As of April 2023, approximately 6.7 million Americans aged 65 years and over suffer from AD [2], and by the end of 2024, this figure is estimated to reach 6.9 million [3]. In the United States, the annual incidence rate of DLB across all age groups is 3.9 per 100,000 individuals, whereas among those aged 65 years and older, the incidence rate increases to 31.6 per 100,000 individuals [4].

In clinical practice, AD and DLB are often misdiagnosed. Studies have shown that among DLB patients determined by autopsy pathology, the misdiagnosis rate is as high as 50%, and the vast majority are diagnosed with AD [5]. This is partly because of their overlapping clinical presentations, which include significant memory decline, aphasia, impaired executive functions, and psychiatric symptoms such as hallucinations, delusions, and impulsivity [6]. Additionally, the pathological changes observed in the brains of patients with AD and DLB can be influenced by other diseases, genetic factors, and coexisting conditions such as amyloid plaques, cerebrovascular lesions, frontotemporal degeneration, and atypical brain atrophy [7]. These factors contribute to the complexity of clinical presentations and increase the likelihood of misdiagnosis.

Misdiagnosis often leads to rapid disease progression due to the lack of timely and effective intervention, especially in the case of DLB [8]. Accurate and early diagnosis is crucial, particularly for pharmacological interventions. Medications designed to ameliorate dementia symptoms have been shown to improve clinical manifestations and slow disease progression [9]. Newer medications, such as disease‐modifying treatments, primarily target patients with mild cognitive impairment (MCI) and early‐stage AD. The FDA‐approved drug lecanemab, which demonstrated significant clearance of amyloid‐beta plaques in the brains of early AD patients during clinical trials in 2023 [10], exemplifies the importance of early and accurate diagnosis for effective treatment. Conversely, misdiagnosis can lead to inappropriate treatment plans; each type of dementia has its recommended medications, and using the wrong drugs can not only be ineffective but may also exacerbate the condition.

Currently, the clinical diagnosis of AD and DLB primarily utilizes various clinical diagnostic criteria based on the patient's symptomatic presentation. Commonly used criteria include the International Classification of Diseases (ICD‐11) [11], Diagnostic and Statistical Manual of Mental Disorders‐V (DSM‐V) [12], National Institute of Age‐Alzheimer's Association (NIA‐AA) 2011 [13], International Working Group‐2 (IWG‐2 [14]), and National Institute of Neurological and Communicative Disorders and Stroke‐Alzheimer's Disease and Related Disorders Association (NINCDS‐ADRDA) [15] for diagnosing AD, and the DSM‐V and the Fourth Consensus Report of the DLB Consortium (2017) [16] for diagnosing DLB. Research indicates that the accuracy of diagnoses based on these symptom‐focused criteria, when combined with neuropsychological testing and neuroimaging results, is approximately 81.6% [17].

Compared with traditional symptomatic diagnosis, although the biological diagnosis of AD has a higher accuracy rate, it cannot be carried out on a large scale due to difficulties in collecting cerebrospinal fluid (CSF) samples from dementia patients and the high hardware configuration requirements for PET scans. At present, there are still no biological diagnostic criteria for DLB.

Over the past few decades, evidence from various fields, including gross anatomical changes, pathological alterations, genetic genomics, and histopathology, has indicated abnormalities in the immune system associated with neurodegenerative diseases. With continuous advancements in technologies such as genome‐wide association studies, single‐cell sequencing, and mass cytometry, increasing high‐resolution immunomic differences have been revealed. These findings underscore the immune system's critical role in the onset and progression of neurodegenerative diseases, suggesting a primary rather than secondary response [18].

Peripheral blood mononuclear cells (PBMCs) are a major component of the human immune system, consisting of key elements of both the innate and adaptive immune responses. Studies [19, 20] have revealed distinctive peripheral immune cell alterations during the progression of neurodegenerative diseases, aiming to highlight differences in immune composition between these diseases and healthy controls (HC). Compared to DLB, more studies have focused on PBMCs in AD. However, conclusions about the peripheral immune profiles among AD, DLB, and HCs vary significantly, and most findings have not been replicated. This variability may be attributed to limitations such as insufficient resolution of flow cytometry, small sample sizes, or varying degrees of neurological damage in study participants.

To provide more objective and precise support for clinical diagnoses and to explore the peripheral immune characteristics of AD and DLB, we utilized time‐of‐flight mass cytometry (CyTOF) with single‐cell resolution to comprehensively analyze the PBMCs from 57 individuals. This approach identified over 1000 immune characteristics. We constructed immune disease models that demonstrated high diagnostic efficacy in identifying diseased samples and distinguishing AD from DLB cases. Our results indicate that peripheral immune characteristics could serve as promising biomarkers to facilitate the clinical diagnosis of AD and DLB.

## 2 Materials and Methods

### 2.1 Patients and Sample Collection

Participants with DLB and AD, along with HC, were recruited from the neurology outpatient clinic of the First Affiliated Hospital, College of Medicine, Zhejiang University. Written informed consent was obtained from all participants, and the study was conducted following the protocol approved by the Clinical Research Ethics Committee of the First Affiliated Hospital, College of Medicine, Zhejiang University (Approval No. IIT20220213B‐R1). The study was registered at ClinicalTrials.gov PRS, with registration number NCT05518409.

The clinical team comprised a chief neurologist, an associate chief psychiatrist, and two neuropsychological assessors, all of whom had undergone standardized training. For clinical diagnostic criteria, patients with DLB and AD first had to meet the criteria for Major Neurocognitive Disorders in the DSM‐V [12]. Subsequently, DLB patients had to fulfill the criteria for Probable DLB outlined in the Fourth Consensus Report of the DLB Consortium (2017) [16], while AD patients had to meet the criteria for Probable AD dementia according to the NIA‐AA workgroups on diagnostic guidelines for Alzheimer's disease [13].

In our study, we collected samples from 57 participants and recorded their general information, administering neuropsychological assessment questionnaires. All participants underwent cranial MRI scans, including axial and coronal views, with hippocampal medial temporal lobe atrophy grading requirements as follows: < 75 years old, grade 2 or higher; ≥ 75 years old, grade 3 or higher. Basic information for the three groups was displayed in color block diagrams (Figure S1A), and baseline data comparisons among the three groups were described in Figure S1B. Peripheral blood samples were collected from all participants and processed into plasma and PBMCs samples.

### 2.2 Peripheral Blood Plasma p‐Tau181 Detection

To measure p‐Tau181 levels in plasma, we used the Human Tau (Phospho) [pT181] ELISA Kit (#KHO0631, Invitrogen, MA, USA). This test utilizes a capture antibody specific for phospho‐Tau (pT181), which is pre‐coated onto the wells of a 96‐well plate. During the initial incubation, standards with known concentrations and test samples are added to the wells, allowing the target antigen to bind to the fixed antibody. Following a wash step to remove unbound substances, a rabbit antibody targeting the same protein is added as a detection antibody, binding to the captured antigen. Following another wash step, horseradish peroxidase (HRP)‐conjugated anti‐rabbit IgG is introduced, forming a complete sandwich complex. After a final incubation and washing to remove unbound enzyme, a 3,3′, 5,5′‐Tetramethylbenzidine (TMB) substrate solution is added. This substrate reacts with the enzyme, producing a color change proportional to the amount of target protein in the samples. The resulting optical density is measured using a standard microplate reader.

### 2.3 Plasma NFL Measurement

We used the human neurofilament light (NFL) ELISA Kit (Novus Cat no: NBP2‐81184, USA) to assess the NFL levels in plasma. First, the necessary strips from the foil pouch were allowed to reach room temperature for 20 min. Wells were assigned wells for standards and samples: 50 μL of various concentrations of the standard were added to the designated standard wells, and 50 μL of each sample was added to the sample wells, leaving the blank wells empty. Subsequently, 100 μL of HRP‐conjugated detection antibody was added to each of the standard and sample wells, except for the blank wells. The plate was covered with a film and incubated at 37°C in a water bath or incubator for 60 min. After incubation, the liquid was removed, and the plate was tapped dry on an absorbent paper. Each well was then filled with 350 μL of wash buffer, allowed to rest for 1 min, and the wash buffer was discarded. This washing step was repeated five times with optional use of a plate washer. Next, 50 μL each of substrate A and B were added to each well, and the plate was incubated in the dark at 37°C for 15 min. Finally, 50 μL of stop solution was added to each well, and the optical density (OD) was measured at a wavelength of 450 nm within 15 min.

### 2.4 CyTOF Sample Processing and Data Analysis

We collected peripheral blood using ethylene diamine tetraacetic acid anticoagulant tubes. PBMCs and plasma were separated using the Ficoll density gradient centrifugation method. Post‐separation, the PBMCS were washed with prechilled fluorescence‐activated cell sorting (FACS) buffer to cleanse the cell pellet. Red blood cells were lysed using acetone cyanohydrin ketal (ACK), and the cells were resuspended in FACS buffer for manual or instrumental counting. The criteria for sample viability included a minimum live cell count of 3 × 10^6^ cells and a viability of no less than 85%.

For staining, 3 × 10^6^ cells from the PBMC samples were isolated and stained for cell viability using cisplatin. Following a wash with FACS buffer, a fragment crystallizable block was applied. The cells were then stained for 30 min using a panel of surface antibodies, consisting of 21 cell surface markers (Table S1), to identify 29 immune cell subgroups (Table S2). Additionally, 21 intracellular signaling molecules were used (Table S1) for intracellular signal labeling. After another wash with FACS buffer, cells were fixed and permeabilized for intracellular antibody staining. DNA staining was performed using a fix and perm buffer and incubated overnight. Finally, the cells were washed with FACS buffer, resuspended in deionized water containing 10% EQ beads, and filtered through a 40 μm cell strainer before CyTOF analysis.

All sample data were decoded, normalized, and processed using FlowJo software to manually exclude fragments and dead cells, retaining only single, live immune cells. Unsupervised clustering of all immune cells was conducted using the phenotyping by accelerated refined community‐partitioning (PARC) [21] algorithm. Cell subgroups were annotated based on their marker expression patterns as shown in the “cluster vs marker” heatmap. Data visualization was facilitated using the t‐distributed stochastic neighbor embedding(t‐SNE) dimensionality reduction algorithm [22].

The subpopulation abundance data of all groups were verified for normality through the Kolmogorov–Smirnov test. If the *p*‐value > 0.05, the data are considered to be normally distributed; if it is less than 0.05, the data are regarded as being non‐normally distributed (Table S3). The comparisons of abundance between different clusters were displayed using dot plots. For the comparison of abundance values between two groups, an independent‐sample *t*‐test (two‐sided) is used when the data of both groups are normally distributed. If the data of one or both groups are not normally distributed, the Mann–Whitney U test is employed instead, with statistically significant *p*‐values (*p < 0.05, **p < 0.01, ***p < 0.001, and if the *p*‐value exceeds 0.05, it is noted in the figure).

Correlations between immune subpopulations across different groups were analyzed using Spearman correlation analysis. Heatmaps displayed the *r*‐values, with * marking subpopulations that showed significant differences, representing *p* < 0.05. In the baseline data of the three groups, the quantitative data were also verified for normality through the Kolmogorov–Smirnov test (Table S3). For the data where all three groups were normally distributed, the one‐way ANOVA analysis was adopted. For those with at least one group of data being non‐normally distributed, the Kruskal–Wallis test was used for analysis. In pairwise comparisons, an independent‐sample *t*‐test (two‐sided) was employed for the quantitative data that were normally distributed. If the data of one or two groups were non‐normally distributed, the Mann–Whitney U test was adopted instead, while categorical data were assessed using the chi‐square test. A *p*‐value < 0.05 was considered statistically significant.

### 2.5 Constructing Disease Immune Feature Matrices and Selecting Optimal Features for Building Disease Models

Peripheral immune cell subtypes were categorized into three levels based on CD45 proportions:

1. Major cell types: T cells, B cells, NK cells, and myeloid cells.
2. Subtypes: CD4, CD8, CD4 TCM (central memory), CD4 TEM (effector memory), CD8 TEMRA (terminally differentiated effector memory), and CD8 TEM.
3. Detailed subtypes: T01–T18, B01–B02, NK01–NK04, and M01–M05.

This categorization results in a matrix of 39 groups of data, with expression values of each marker in 29 subtypes (41 × 29), yielding a total of 1228 immune feature data points after normalization.

To develop a disease diagnostic model, data were segmented into three groups: AD/HC, DLB/HC, and AD/DLB. Spearman correlation coefficients between the 1228 immune features and the corresponding groups were calculated separately. Significant features were sorted by *p*‐values and the most correlated immune features for each group were selected and visualized using a heatmap to display the r and *p*‐values. These significant immune features may represent differences in subtypes at different levels or the expression values of a specific marker within a subtype.

The optimal differentiating immune features were loaded into the immunological elastic net (iEN) model and iterated 100 times to compute the disease model. Initially, the *p*‐values of Spearman correlation coefficients were sorted and transformed using −log10. The cumulative value of −log10 (*p*‐value) was then computed. By calculating the first and second derivatives, trends in the data were observed, with inflection points identified where the second derivative equaled zero. All immune features before these inflection points were loaded into the iEN model.

### 2.6 Immunological Elastic Net (iEN) Model

To establish an accurate disease prediction model, we adopted iEN model [23]. This machine learning approach is well‐suited for processing high dimensional immunological data due to its robust exploratory capabilities. The model was iterated 100 times using a ten‐layered cross‐validation scheme. During each iteration, the model optimized and evaluated a randomly selected subset of data. After completing 100 iterations, the model generated *p*‐values based on the Mann–Whitney U test and the area under the curve (AUC) values, indicating the model's predictive capacity and efficacy.

## 3 Results

### 3.1 Single‐Cell Immune Profiling of PBMCs in AD, DLB, and HC

The study included 57 participants: 14 patients with AD (6 males, 8 females), 25 patients with DLB (11 males, 14 females), and 18 HC (6 males, 12 females). The mean ages were 76.79 ± 6.36 years for the AD group, 72.12 ± 7.76 years for the DLB group, and 73.72 ± 7.29 years for the HC group. Mini‐mental state examination (MMSE) scores were 12.36 ± 7.00 for AD, 16.80 ± 7.47 for DLB, and 27.39 ± 2.55 for HC. Demographic variables such as gender, age, marital status, education, residence, smoking, alcohol consumption, family history, duration of residence, tea/coffee consumption, and MMSE scores were compared among the three groups, with significant differences observed only in MMSE scores (Figure S1A,B).

**FIGURE 1:**
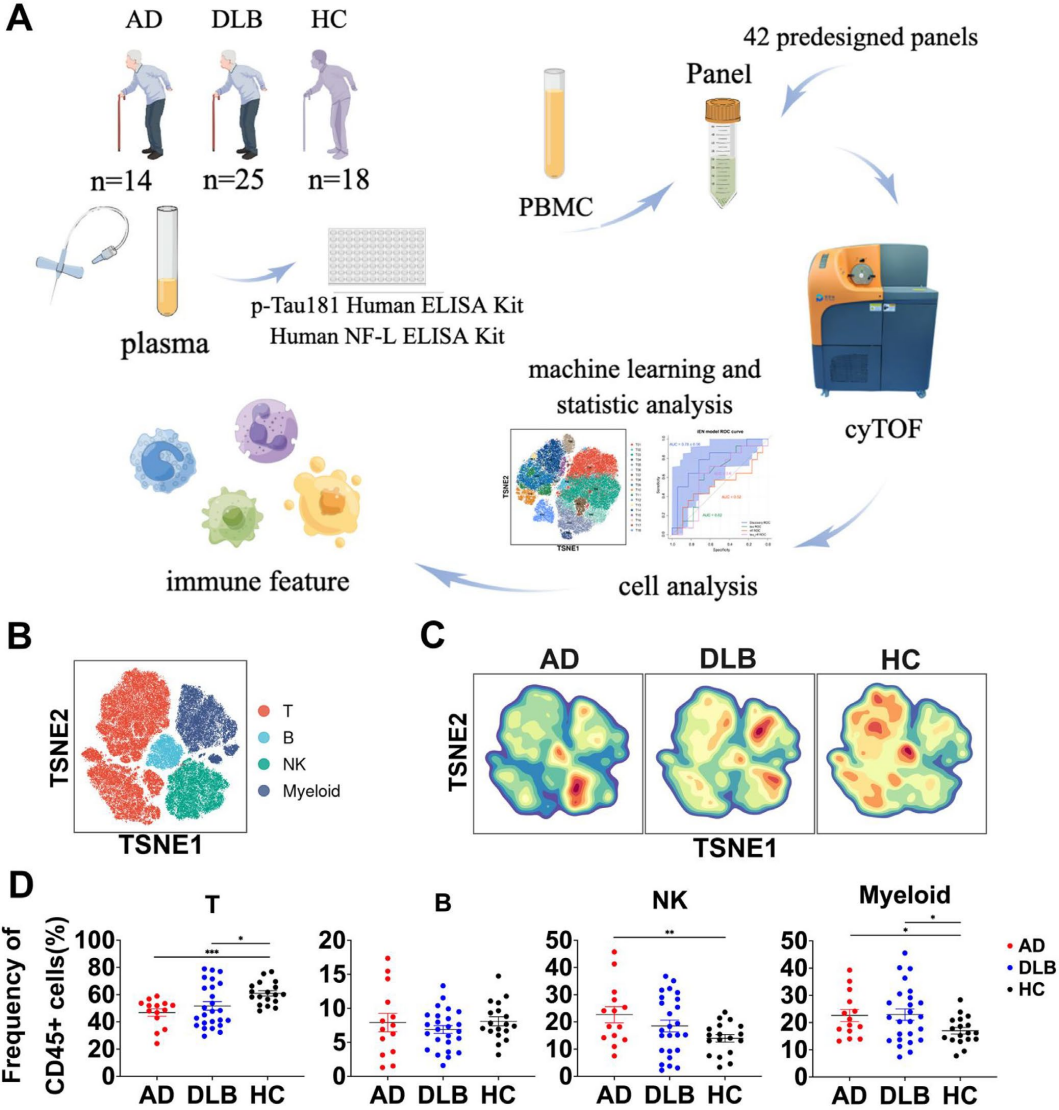
Single‐cell CyTOF analysis reveals the primary peripheral immune components in AD, DLB patients, and HC. (A) Experimental design and data analysis workflow for single‐cell CyTOF by Figdraw. (B)A t‐SNE plot of 60,000 randomly sampled immune cells from the three groups, where different colors represent various major immune cell subpopulations. (C) Contour plots of cell density distributions in AD, DLB patients, and HCs. (D) Comparison of the proportions of major immune cell subpopulations among the groups. Statistical analysis was performed using the independent‐samples *t*‐test or the Mann–Whitney U test. (D) *p < 0.05, **p < 0.01, ***p < 0.001.

To investigate the peripheral immune differences between AD and DLB patients, single‐cell CyTOF analysis was performed using a predesigned antibody panel (Table S1) on collected peripheral blood samples (Figure 1A). After data preprocessing, four major immune subsets (T cells, B cells, NK cells, and myeloid cells) were identified using the PARC clustering method. The t‐SNE plots showed that T cells constituted the largest proportion (53.02%) of the identified immune subsets, followed by myeloid cells (21.21%) and NK cells (18.30%) (Figure 1B). Moreover, distinct density distributions of these immune subsets were observed among different sample groups (Figure 1C), with high‐density regions primarily concentrated in NK cells for AD patients, myeloid cells for DLB patients, and T cells for HC. Frequency comparisons of the major immune subsets confirmed these observations (Figure 1D). Specifically, AD patients exhibited lower levels of T cells (*p* < 0.001) and higher levels of NK cells (*p* = 0.008) and myeloid cells (*p* = 0.024) compared to HC, corroborated by the density plots. DLB showed lower levels of T cells (*p* = 0.030) and higher levels of myeloid cells (*p* = 0.031) compared to HC, also demonstrated in the density plots.

### 3.2 Single‐Cell Immune Profiling of T Cells

**FIGURE 2:**
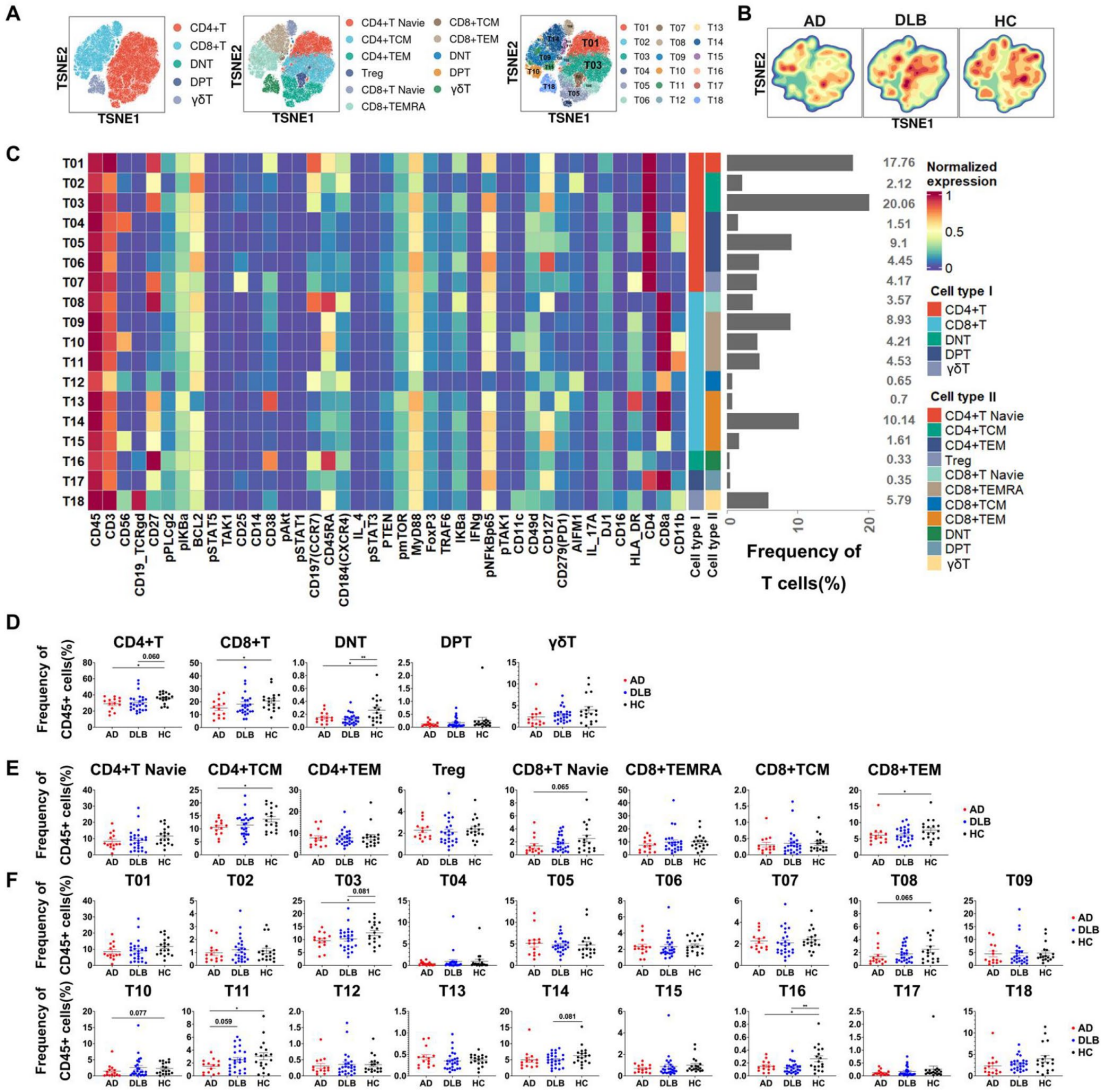
Characteristics of peripheral blood T lymphocytes in AD, DLB patients, and HC. (A) t‐SNE plots of T cells from the AD, DLB, and HC groups, colored by identified T cell clusters and divided into three tiers. (B) Contour plots of T cell density distribution in AD, DLB patients, and HCs. (C) Heatmap of normalized median expression of the identified 18 T cell groups. The gray bar on the right indicates the relative frequency of T cell groups. Functional cell types are marked with colors on the right and annotated. (D) Comparison of the percentages of identified CD4+ T cells, CD8+ T cells, T cells, DNT cells, and DPT cells among different groups. (E) Comparison of the percentages of identified functional subgroups of CD4+ T cells and CD8^+^ T cells among different groups. (F) Comparison of the percentages of the identified 18 T cell subgroups among different groups. Statistical analysis was performed using the independent‐samples *t*‐test or the Mann–Whitney U test. (D–F) *p < 0.05, **p < 0.01, ***p < 0.001. *p*‐values greater than 0.05 are noted with specific numerical values in the figure.

All T cells were clustered into 18 subsets, including seven CD4^+^ T (59.17%), eight CD8^+^ T (34.35%), one γδT (5.79%), one double negative T (DNT, 0.33%), and one double positive T (DPT, 0.35%) cell subsets (Figure 2A,C). Based on CCR7 and CD45RA markers, these T cells were further categorized into 11 subgroups (Table S2). Among them, CD4^+^ naïve (30.02%, T01), TCM (37.50%, T02–T03), and TEM (25.45%, T04–T06) T cells made up the majority of CD4^+^ T cells; CD8^+^ recently activated TEM T cells (CD8^+^ TEMRA, 51.44%, T09–T11) and TEM cells (CD8^+^ TEM, 36.24%, T13–T15) were predominant in CD8^+^ T cells. The t‐SNE plots revealed significantly different high‐density distribution areas across groups (Figure 2B).

Statistical analysis confirmed significantly enriched frequencies of CD4^+^ TCM and DNT in HC compared to AD and DLB groups (Figure 2D,E). T03 and T11 cells were significantly more abundant in HC than in AD patients (Figure 2F).

In the first tier of T cells, AD patients exhibited significantly lower proportions of CD4^+^ T cells (*p* = 0.011), CD8^+^ T cells (*p* = 0.044), and DNT cells compared to HC (Figure 2D). DNT levels in DLB were significantly lower than in HC (*p* = 0.003), with a downward trend in CD4^+^ T cells (*p* = 0.060) (Figure 2D). In the second tier, differences among the three groups were less pronounced. Compared with HC, the CD4^+^ TCM cells (*p* = 0.028) and CD8^+^ TEM cells (*p* = 0.049) in patients with AD were significantly decreased, and there was a downward trend in CD8^+^ T Naive cells (*p* = 0.065). In the third tier, the T03 subgroup of CD4^+^ TCM in AD was significantly lower than in HC (*p* = 0.035), characterized by a (CD27^+^pNFkBp65^+^AIFM1^−^) phenotype. Among CD8^+^ TEMRA cells, the T11 subgroup was significantly lower in AD compared to HC (*p* = 0.029), characterized by (CD27‐CD56‐CD11b+) phenotype, the T10 subgroup showed a downward trend (*p* = 0.077), and its characteristics were (CD27^−^ CD56^+^ CD11b dim). (Figure 2F). In DLB, the T03 subgroup from CD4^+^ TCM showed a downward trend compared to HC (*p* = 0.081), and the T14 subgroup in CD8^+^ TEM was slightly lower in DLB compared to HC, but not significantly (*p* = 0.081) (Figure 2F). Differences in T cell subgroups between AD and DLB were minimal, with the T11 subgroup from CD8^+^ TEMRA showing a trend of lower levels in AD compared to DLB (*p* = 0.059) (Figure 2F).

### 3.3 Distinct Alterations Identified in B, NK and Myeloid Subsets

**FIGURE 3:**
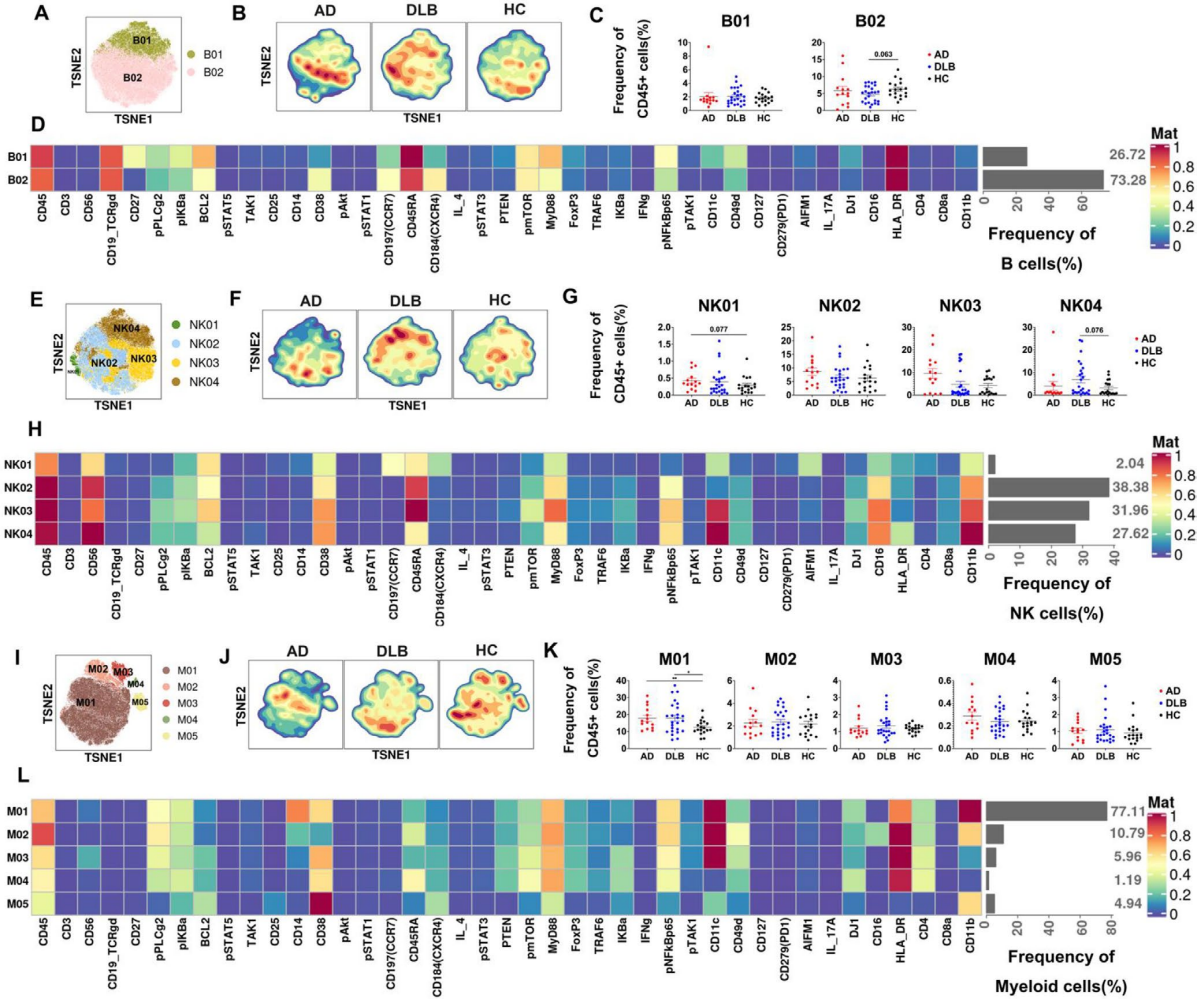
Characteristics of peripheral blood B lymphocytes, NK cells, and myeloid cells in AD, DLB patients, and HC. (A, E, I) t‐SNE plots of B cells (A), NK cells (E), and myeloid cells (I) from the AD, DLB, and HC groups, colored by identified subgroups of B cells, NK cells, and myeloid cells, respectively. (B, F, J) t‐SNE plots of cell density distribution for B cells (B), NK cells (F), and myeloid cells (J) across the groups. (C, G, K) Comparison of the frequencies of major subgroups of B cells (C), NK cells (G), and myeloid cells (K) among different groups. (D, H, L) Heatmaps of normalized median expression for two identified B‐cell clusters (D), four identified NK‐cell clusters (H), and five identified Myeloid‐cell clusters (L). Bar graphs showing the relative frequencies of B‐cell clusters, NK‐cell clusters, and myeloid‐cell clusters are displayed with a gray bar on the right. Statistical analysis was performed using the independent‐samples *t*‐test or the Mann–Whitney U test. (C, G, K) *p < 0.05, **p < 0.01, ***p < 0.001. *p*‐values greater than 0.05 are noted with specific numerical values in the figure.

Using t‐SNE plots, we identified two distinct B cell subsets (Figure 3A). Based on the expressions of surface markers, B01 was identified as memory B cells, and B02 as naïve B cells, with B02 comprising the majority proportion (73.28%) (Figure 3D). The density distribution of B cells (Figure 3B) indicates that, compared to HC, AD exhibits a higher density in certain areas of B02, while DLB shows higher density areas in both B01 and B02, though the distribution intervals vary slightly. HC does not exhibit significant high‐density areas. The boxplots of B cell subsets across the three groups (Figure 3C) reveal that the main differences in B cell abundance are observed between DLB and HC, with a trend toward lower abundance of B02 in DLB patients compared to HC (*p* = 0.063).

The t‐SNE plot illustrates that NK cells are divided into four subsets: NK01 (2.04%), NK02 (38.38%), NK03 (31.96%), and NK04 (27.62%) subsets being the most prominent (Figure 3E,H). The density distribution of NK cells (Figure 3F) shows that, compared to HC, the overall distribution of NK cells in AD patients is more dispersed, with small high‐density areas in NK03. In DLB patients, increased expression is observed in certain areas of NK02 and NK04. Dot plots of the NK01‐NK04 subsets (Figure 3G) indicate that, compared to HC, the proportion of the NK01 subset in AD is significantly higher (*p* = 0.077). This suggests that the elevated levels of NK cells in AD are primarily due to an increase in the NK01 subset (pNFkBp65^−^ CD16^−^ CCR7^+^). In the DLB population, there is an increased abundance of the NK04 subgroup compared to HC (*p* = 0.076). However, no significant differences or trends were observed between DLB and HC in the NK01–NK03 subgroups.

The t‐SNE plot for myeloid cells (Figure 3I) reveals five subsets: M01 representing classical monocytes, M02 nonclassical monocytes, M03 conventional dendritic cells (cDC), M04 plasmacytoid DCs (pDC), and M05 basophils. The M01 subset is the largest, constituting 77.11% of the entire myeloid cell population (Figure 3L). The t‐SNE density map for myeloid cells (Figure 3J) shows that compared to HC, AD patients primarily exhibit high‐density regions in M01. Similarly, DLB patients show high‐density distributions in M01, though the areas differ from those in AD. Boxplots of myeloid cells for AD, DLB, and HC populations (Figure 3K) indicate that the overall proportion of myeloid cells in AD is notably higher than in PC (*p* = 0.024) (Figure 1C). Analyzing myeloid cell subsets (Figure 3K), the significant difference in AD may be attributed to the higher proportion of the M01 subset compared to HC (*p* = 0.009), while no significant differences or trends were observed in other subsets (M02–M05). Similarly, the overall level of myeloid cells in DLB is significantly higher than in HC (*p* = 0.031) (Figure 1C), primarily due to a significant increase in the M01 subset compared to HC (*p* = 0.022) (Figure 3K).

### 3.4 The Correlation Between Peripheral Immune Subsets Varies Among Different Disease and Healthy Populations

**FIGURE 4:**
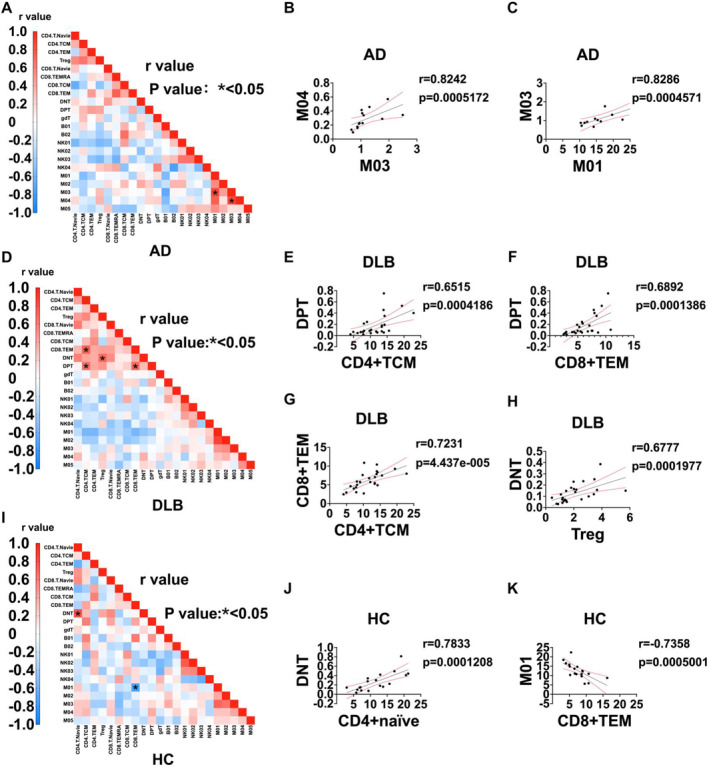
Associations among peripheral immune subgroups in AD, DLB patients, and HC. (A, D, I) Heatmaps of Pearson correlation coefficients between immune cell subgroups within each group for AD, DLB, and HC, with *p*‐values less than 0.05 marked with an asterisk in the figure. (B, C, E–H, J, K) Correlations and 95% confidence interval (CI) regression lines for the percentages of different immune cell subgroups within each group. Statistical analyses were conducted using Pearson correlation. (A, D, I) *p < 0.05. (B, C, E–H, J, K) *p*‐values and r‐values are noted with specific numerical values in the figure.

To elucidate the correlation among peripheral immune subsets in AD, DLB, and HC populations, we conducted a Spearman correlation analysis on the abundance values of immune cells from each population. The results are presented in a heatmap displaying the correlation coefficients (r values), highlighting significant differences among the immune subsets. A total of 22 subsets were included in the analysis. The findings demonstrate that interactions among immune subsets in the AD group are predominantly concentrated in myeloid cells, with M03 showing significant positive correlations with both M01 and M04 (Figure 4A). In the DLB group, CD4^+^ TCM exhibits a positive correlation with CD8^+^ TEM, and DPT cells are positively correlated with CD4^+^ TCM, CD8^+^ TEM, and regulatory T cells (Tregs) (Figure 4B). In the HC cohort, CD4^+^ T naïve cells are positively correlated with DNT, while CD8^+^ TEM are negatively correlated with M01 (Figure 4C).

### 3.5 Construction of High Diagnostic Efficacy Disease Models Using Peripheral Single‐Cell Immune Features

To construct diagnostic models for diseases using single‐cell peripheral immune features, we aimed to identify immunological differences and compare diagnostic efficacy with peripheral plasma p‐Tau181 and NFL. We incorporated the most characteristic peripheral immune features of the three groups into the iEN model. The results from all three group models effectively distinguished between the two types of individuals within each group. The differential single‐cell peripheral immune features demonstrated strong diagnostic performance within the iEN model framework. We compared the iEN model's diagnostic efficacy, represented by receiver operating characteristic (ROC) curves, with peripheral plasma p‐Tau181, NFL, and the combined diagnostic efficacy of p‐Tau181 + NFL by comparing AUC values. We found that the diagnostic efficacy of the immune model, composed of peripheral single‐cell immune features, significantly exceeds that of peripheral plasma p‐Tau181, NFL, and the combined p‐Tau181 + NFL diagnostic efficacy.

### 3.6 AD/HC Model

**FIGURE 5:**
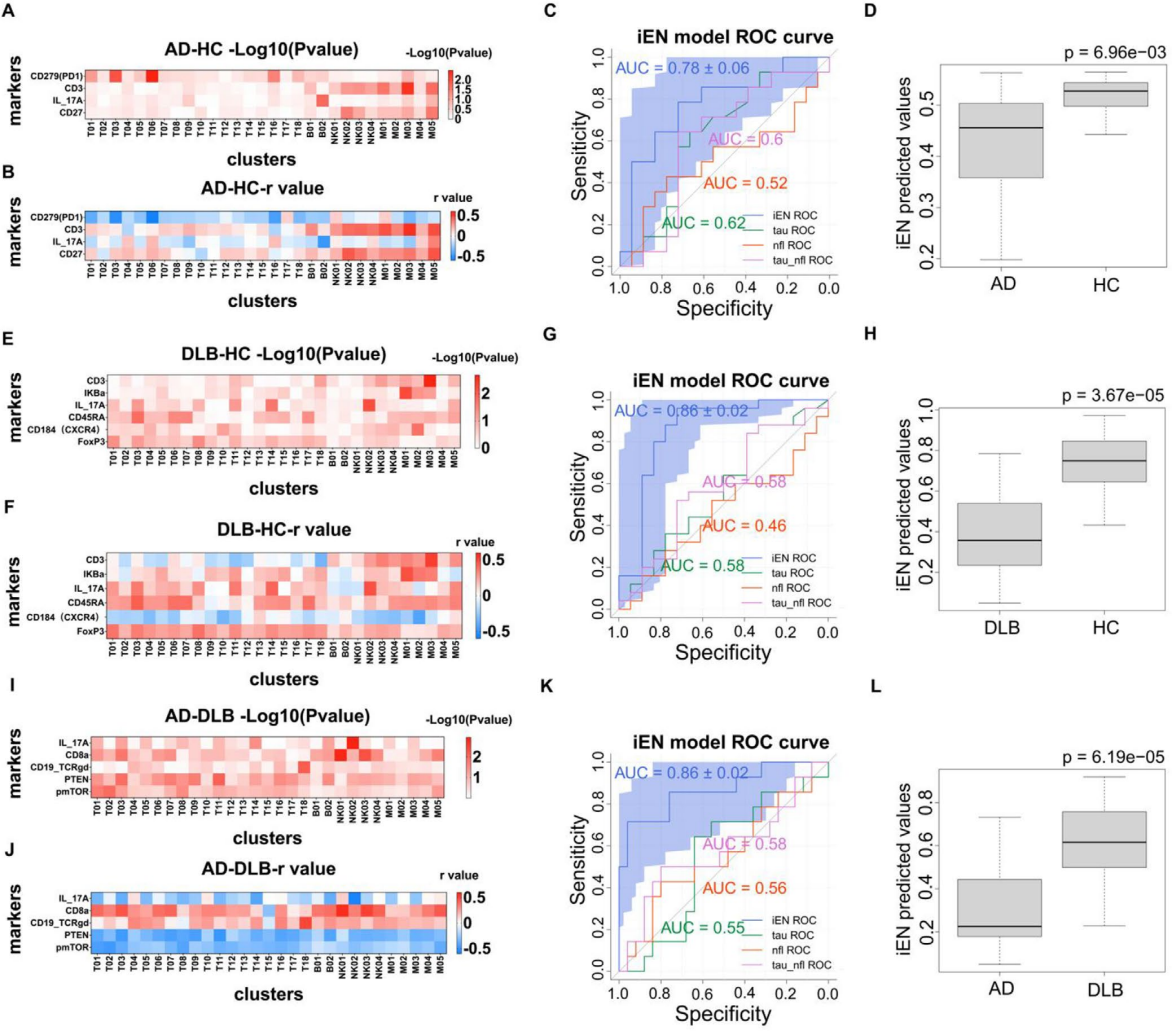
Construction of efficient iEN disease diagnostic models using dominant immune features from any two of the AD/HC, DLB/HC, and AD/DLB groups. (A, B, E, F, I, J) Disease models formed by the AD group and HC group (A, B), the DLB group and HC group (E, F), and the AD group and DLB group (I, J), featuring heatmaps of Spearman correlation coefficients between the most differential markers and each cell subgroup. (C, G, K) ROC curves for the iEN model‐predicted AD/HC (C), DLB/HC (G), and AD/DLB (K) disease models and peripheral plasma pTau‐181, NFL, along with AUC values, demonstrating high diagnostic efficacy of the three disease models. (D, H, L) Comparison of iEN predicted values between two populations within the three disease models, with *p*‐values calculated using the Mann–Whitney U test.

In the AD/HC group, we identified the most divergent eight immune features, ranked by correlation strength: T, T06CD279 (PD1), M03CD3, T03CD279 (PD1), B02IL_17A, M05CD3, M01CD3, and M05CD27. Among these, T represents a cell subgroup, while the other seven features are marker expressions on specific subgroups. The abundance of T cell subgroups in AD was lower than in HC (Figure 1C). The seven immune features are concentrated in four markers: CD279 (PD1), CD3, IL_17A, and CD27. We plotted the Spearman correlation r‐values and *p*‐values of these four markers with all subgroups in a heatmap (Figure 5A,B) to represent the performance of these significant immune markers across all subgroups. We found that CD279 (PD1) exhibited higher differential expression in T06 (CD4^+^ TEM) and T03 (CD4^+^ TCM) but not significantly in other subgroups (Figure 5A). Expression levels of CD279 (PD1) in AD were significantly higher in both T06 and T03 compared to HC (Figure S2A), and generally higher in other subgroups compared to HC, showing unidirectional expression. CD3 in AD patients showed lower expression in M03, M05, and M01 compared to HC (Figure S2A), with CD3 expression in most subgroups of AD patients being lower than in HC, exhibiting unidirectional expression. IL_17A in AD patients showed bidirectional expression across all subgroups, with expressions both lower and higher than in HC. The most significant differential expression was observed in the B02 subgroup, where expression levels were higher than in HC (Figure S2A). CD27 exhibited bidirectional expression, with the most divergent subgroup being M05, where AD's M05CD27 expression was lower than in HC (Figure S2A).

We incorporated the eight differential immune features from the AD/HC group into the iEN model. After 100 iterations, the model generated *p*‐values based on the Mann–Whitney U test (Figure 5D) and ROC curves (Figure 5C). The AUC of the AD/HC model was 0.78, indicating excellent diagnostic performance. Comparison of iEN predicted values between AD and HC yielded a *p*‐value of 6.96 × 10^−3^, as depicted by boxplot (Figure 5D), illustrating the model's ability to effectively distinguish between the two groups. Additionally, we utilized peripheral plasma p‐Tau181, NFL, and p‐Tau181 + NFL data from both groups for diagnosis, yielding AUC values of 0.62, 0.52, and 0.6, respectively—all lower than the AUC value of the single‐cell immune model for AD/HC. This indicates that the diagnostic efficacy of the single‐cell immune model for AD/HC surpasses that of peripheral plasma p‐Tau181 and NFL.

### 3.7 DLB/HC Model

Using the same methodology, the diagnostic efficacy of DLB/HC and AD/DLB models was evaluated using single‐cell immune data. Eleven most divergent immune features were identified in the DLB/HC model, including M03CD3, M01 IKBa, NK02IL_17A, T03CD45RA, M02CD184 (CXCR4), T14IL_17A, T16, T07CD45RA, T06CD45RA, T08FoxP3, and T17CD45RA, of which only T16 represented an immune subgroup and the rest were expressions of markers across different subgroups. The abundance of T16 was lower in DLB than in HC (Figure 2F).

Divergent markers included CD3, IKBα, IL_17A, CD45RA, CD184 (CXCR4), and FoxP3 (Figure 5E). CD3 exhibited bidirectional expression (Figure 5F); compared with the HC subgroup, it was highly expressed in some DLB subgroups and lowly expressed in others. Specifically, in the M03 subgroup, CD3 expression was significantly lower in DLB than in HC (Figure S2B). IKBα, IL_17A, CD45RA, CD184 (CXCR4), and FoxP3 exhibited unidirectional expression in the DLB/HC model (Figure 5F), with CD184 (CXCR4) expression levels generally higher across all subgroups in DLB compared with HC (Figure 5F), which was significantly higher in the M02 subgroup (Figure S2B). However, IKBα, IL_17A, CD45RA, and FoxP3 expression levels were lower across all subgroups in DLB than in HC (Figure 5F), with IKBα in the M01 subgroup; IL_17A in NK02 and T14 subgroups; CD45RA in T03, T07, T06, and T17 subgroups; and FoxP3 in the T08 subgroup being significantly lower in DLB than in HC (Figure S2B).

Regarding the iEN model, the AUC of the DLB/HC model was 0.86 (Figure 5G), significantly higher than the diagnostic efficacy of peripheral plasma p‐Tau181 (0.58), NFL (0.46), and p‐Tau181 + NFL (0.58). A comparison of iEN‐predicted values between DLB and HC yielded a *p*‐value of 3.67 × 10^−5^ (Figure 5H).

### 3.8 AD/DLB Model

In the diagnostic models constructed for AD and DLB, the most divergent immune features include NK02IL_17A, NK01CD8a, NK03CD8a, T18CD19_TCRgd, T11PTEN, and T02pmTOR (Figure 5I). Five markers were divergent, including IL_17A, CD8a, CD19_TCRgd, PTEN, and pmTOR, all exhibiting unidirectional expression (Figure 5J). The expression levels of CD8a and CD19_TCRgd across all subgroups were lower in AD than in DLB. CD8a expression in NK01 and NK03 subgroups was significantly lower in AD than in DLB. CD19_TCRgd expression in the T18 subgroup was significantly lower in AD than in DLB. However, IL_17A expression in the NK02 subgroup, PTEN in the T11 subgroup, and pmTOR in the T02 subgroup were significantly higher in AD than in DLB (Figure S2C).

The diagnostic efficacy of the AD/DLB iEN model was also excellent, with an AUC of 0.86, surpassing the diagnostic efficacy of peripheral plasma p‐Tau181 (0.55), NFL (0.56), and p‐Tau181 + NFL (0.58) (Figure 5K). Comparison of iEN‐predicted values between AD and DLB yielded a *p*‐value of 6.19 × 10^−5^, enabling effective differentiation diagnosis (Figure 5L).

### 3.9 Gender‐Specific Immune Responses and Gender Influence on AD/HC and DLB/HC Models

To investigate the impact of gender on model efficacy, AD/HC and DLB/HC models were divided based on gender. Initially, the AD/HC model was classified into two groups: [Male AD (6)+Male HC (6) vs. Female AD (8)+Female HC (12)] and [Male AD (6)+All HC (18) vs. Female AD (8)+All HC (18)]. After reclassifying the samples and conducting the Spearman coefficient test on 1228 immune characteristics with the group, *p*‐values changed significantly, indicating the influence of gender.

**FIGURE 6:**
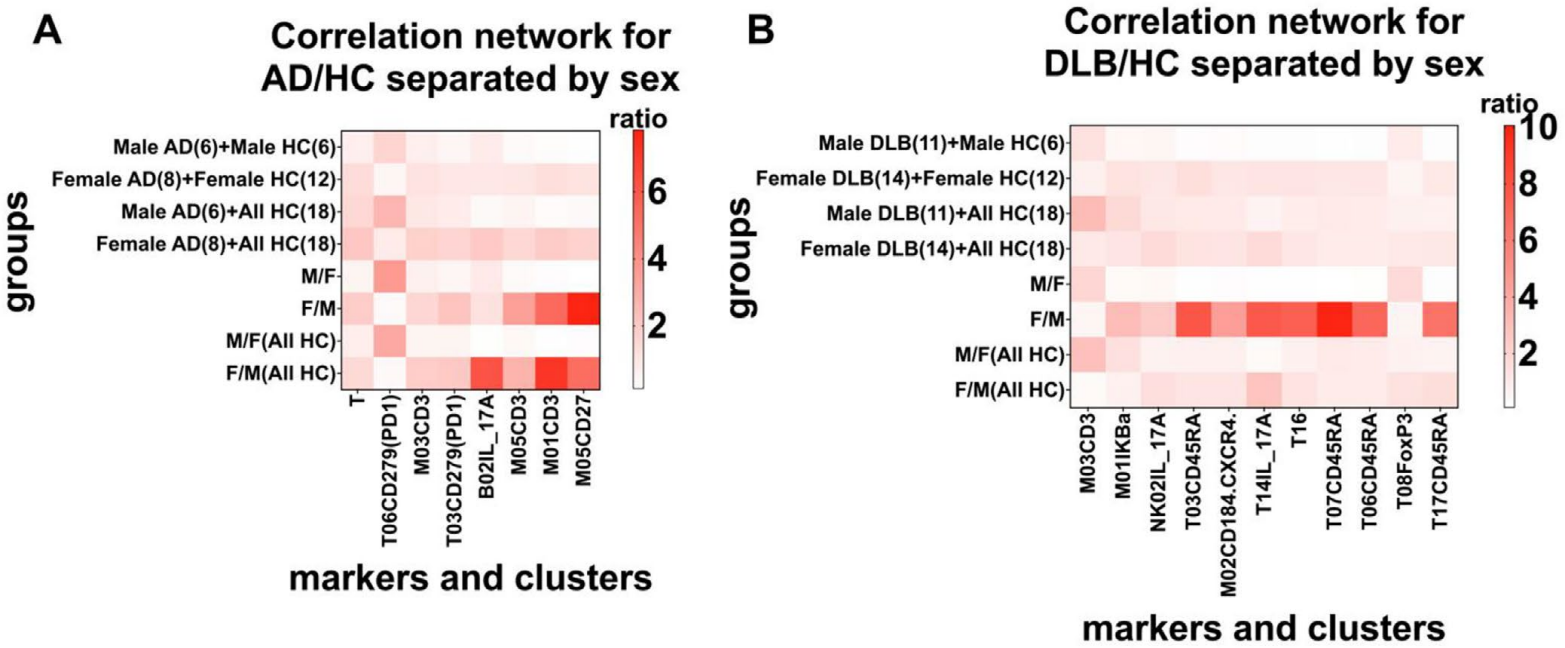
Gender‐specific immune responses in AD. (A and B) Recombined disease models were formed by the AD group and HC group (A), and the DLB group and HC group (B), featuring heatmaps of Spearman correlation coefficients between differential markers and subgroups across various groups composed of different genders.

In the first group [Male AD (6)+Male HC (6) vs. Female AD (8)+Female HC (12)], the male group (Male AD (6)+Male HC (6)) encompassed all male immune characteristics from the AD/HC model, uninfluenced by female characteristics. After analyzing the *p*‐values derived from Spearman correlations of the male and female groups and comparing them with the entire sample data from the AD/HC model, a significant gender influence was noted in two groups. The ‐log10 (*p*‐value) of the originally significant differential eight immune characteristics: T, T06CD279 (PD1), M03CD3, T03CD279 (PD1), B02IL_17A, M05CD3, M01CD3, M05CD27 changed substantially (Figure 6A). The new *p*‐values of the eight immune characteristics were compared, which revealed that the male contribution was primarily in CD279 (PD1) expression, particularly in the T06 subgroup, in the AD/HC model. Conversely, the female contribution was mainly in the expression of CD3 in the M01 and M05 subgroups and CD27 in the M05 subgroup. The second group [Male AD (6)+All HC (18) vs. Female AD (8)+All HC (18)], involved supplementing the HC count from the first group. Similarly, *p*‐values changed significantly. A heatmap of the ‐log10 (*p*‐value) of both categories shows that even with the addition of female HCs in the [Male AD (6)+All HC (18)] group, the *p*‐value trend for CD279 (PD1) remained consistent with the [Male AD (6)+Male HC (6)] group (Figure 6A). A similar result was obtained for the [Female AD (8)+All HC (18)] and [Female AD (8)+Female HC (12)] groups, with consistent trends in CD3 and CD27 (Figure 6A), indicating gender‐specific immune responses in AD patients.

Next, by employing a subdivision strategy similar to that of AD/HC, the DLB/HC cohort was divided into four groups: [Male DLB (11)+Male HC (6)], [Female DLB (14)+Female HC (12)], [Male DLB (11)+All HC (18)], and [Female DLB (14)+All HC (18)]. After comparing the [Male AD (6)+Male HC (6)] and [Female AD (8)+Female HC (12)] groups (Figure 6B), it was found that the immune characteristics that differed (M01 IKBa, T03CD45RA, M02CD184 (CXCR4), T14IL_17A, T16, T07CD45RA, T06CD45RA, and T17CD45RA) were predominantly contributed by the female group (F/M column). However, these differences were not evident in the [Male DLB (11)+All HC (18)] and [Female DLB (14)+All HC (18)] groups, indicating an absence of gender‐specific immune responses in DLB.

## 4 Discussion

PBMCs constitute several classes of peripheral immune cells in the human body and are involved in the pathogenesis and progression of neurodegenerative diseases [24, 25]. The present study exploited CyTOF technology to delineate the peripheral immune atlas at the single‐cell resolution for two common neurodegenerative diseases, AD and DLB, revealing distinctive differences in cell abundance between the diseased samples and healthy donors (Figures 1D, 2D–F, and 3C,G,K).

Particularly, the distribution of peripheral immune cells in AD patients is consistent with previous reports. An animal experiment [26] demonstrated that the decline in CD4^+^ T cell levels started in the early stages of AD and became more pronounced with increasing age and disease severity, consistent with our results. Analysis of the peripheral blood of mice by flow cytometry revealed that the proportion of CD3^+^ cells in 3xTg AD mice was significantly lower than that in WT mice. Another study found that [27] CD3^−^CD14^+^ monocytes (corresponding to M01 + M02 in the current study) and NK cells increased steadily as the disease worsened in AD patients compared with HC. The proportions of CD4^+^ T cells and CD4^+^ TCM were lower in patients with severe AD than in HC. CD8^+^ T cell levels decreased gradually at all stages of AD, with a marked decline in severe AD. These differences in immune subgroups between severe AD and HC align with our experimental results, indicating that the severity of AD significantly affects the proportions of peripheral immune cell subgroups.

However, some small‐sample studies [20, 28] are different from our conclusions. The potential reasons for these differences are as follows. First, the sample sizes in these studies were relatively small, and the enrolled populations were in the early stages of AD or even at the MCI stage, with very low CDR scores. Our sample size was larger than that of previous studies, and the enrolled AD patients were in the severe stage of AD with lower MMSE scores. Second, the age of the subjects in previous studies was generally lower than in our study, resulting in a lesser influence of aging; however, the gender ratios of the populations were uneven.

We believe that the subjects in previous studies may have been influenced by aging. The average age of AD patients in our study was 76.79 years, generally higher than that in the aforementioned studies. Although no age difference was found between AD patients and the HC group in the present study, the impact of aging may be greater in AD patients than in the HC group, affecting the expression of immune cells in PBMCs. Additionally, AD patients in our study were more severely affected, with an average MMSE score of 12.36, and an educational level of high school or above. Studies [29] have found that the downregulation of CCR7 is related to the decline of cognitive ability, which is a characteristic of AD patients in a more severe pathological stage. Our results show that the subgroups with downregulated CCR7 account for 66.58% of all T cells (Figure 2C), further indicating that our AD patients were in a more severe condition.

We also identified the relevant immune subgroups that showed altered proportion between DLB patients and healthy donors. Notably, studies exploring peripheral immune subgroups in DLB are relatively few. A previous study [19] used flow cytometry to detect peripheral PBMCs in AD, DLB, and HC groups and found that the proportion of CD4^+^ T cells was significantly lower in DLB than in AD. In addition, no difference in the proportions of CD8^+^ T and CD8^+^ TEMRA cells was found between DLB and HC. These results are consistent with our findings. Changes in peripheral T cell subgroups in DLB indicate a shift toward an aging adaptive immune system in DLB.

The biological diagnosis of AD has received extensive attention in recent years. The NIA‐AA introduced the β‐amyloid deposition, pathologic tau, and neurodegeneration (ATN) diagnostic system in their 2018 AD biomarker‐based diagnostic framework [30]. This system utilizes CSF quantitative analysis of pathogenic proteins or brain positron emission tomography (PET) scans to objectively diagnose AD, thereby reducing the subjectivity associated with symptom‐based diagnoses. AD patients can undergo CSF collection to test for amyloid β‐protein (Aβ) 42 or the Aβ42/40 ratio, as well as phospho‐Tau (p‐Tau) levels, or receive amyloid and Tau PET scans. By measuring the uptake of specific tracers, this approach allows for a biological diagnosis by clarifying the distribution and quantity of pathogenic proteins [30]. This method is considered the gold standard for diagnosing AD due to its objectivity, and because CSF and PET scans, targeting the central nervous system (CNS), are the closest biological samples to brain tissue, besides brain autopsy slices. Research shows that the accuracy of this diagnostic method can reach 82.7% [17]. However, due to limitations, such as patient compliance, high testing costs, and the need for sophisticated hospital equipment, it is not yet feasible for large‐scale implementation. Currently, there is no corresponding biological diagnostic standard for DLB.

In recent years, the biological diagnosis of AD and other dementias has evolved continuously. The Alzheimer's Association updated its clinical guidelines in 2024, introducing fluid biomarkers as a new method for diagnosing AD [31]. This technique measures levels of Aβ42/40, p‐Tau181, or 217 in patients' peripheral blood, reflecting the presence of these pathogenic substances in the CNS to achieve diagnostic purposes. The diagnostic efficacy of plasma p‐Tau181 and NFL in differentiating AD from other types of dementia is comparable to that of CSF and demonstrates significant discriminative power. Multiple studies have shown that p‐Tau181 can effectively distinguish between AD, DLB, and other types of dementia, while NFL exhibits differentially elevated expression across various types of dementia [32, 33, 34]. The use of fluid biomarkers reduces the risk associated with sampling and is readily accepted compared to CSF‐based biological diagnostics. However, this diagnostic approach requires further validation through large‐scale, prospective studies using standardized protocols and the establishment of abnormal value thresholds for different populations [34].

In view of the current diagnostic status of AD and DLB, we took advantage of the high resolution of cyTOF technology and attempted to establish a disease diagnosis model using single‐molecule immunotechnology. Compared with CSF samples, we have the following advantages in using PBMC samples to distinguish between AD and DLB: (1) Peripheral blood samples are less difficult to collect. However, the collection of CSF is extremely difficult, especially for patients with dementia such as AD and DLB. Their condition significantly reduces the success rate of collection. Moreover, compared with peripheral blood collection, lumbar puncture for CSF collection is more invasive to the human body, and the proportion of patients and their guardians who consent to CSF collection is relatively small. (2) PBMC can reflect the immune status of the whole body and is not affected by the blood–brain barrier. Many studies, including those on AD, have pointed out that the pathogenesis of AD, DLB, and other diseases is related to many factors, including oxidative stress, inflammation, and changes in immune status. These changes can affect the human immune status and thus manifest abnormalities in PBMC. In contrast, CSF can only reflect the characteristics of the CNS due to the physical isolation of the blood–brain barrier. In the subsequent analysis, the iEN model we used was applied to establish a disease diagnosis model based on extensive immune feature data. Through Spearman correlation analysis, we integrated predominant immune feature data into the iEN model, which, after multiple iterations, resulted in the establishment of three disease models: AD/HC (AUC: 0.78_AD/HCmodel_ vs. 0.62_p‐tau181_ vs. 0.52_NFL_ vs. 0.6_p‐tau181+NFL_), DLB/HC (AUC: 0.86_DLB/HCmodel_ vs. 0.58_p‐tau181_ vs. 0.46_NFL_ vs. 0.58_p‐tau181+NFL_), and AD/DLB (AUC: 0.86_AD/DLBmodel_ vs. 0.55_p‐tau181_ vs. 0.56_NFL_ vs. 0.58_p‐tau181+NFL_). It was found that the immune models constructed from single‐cell peripheral immune features exhibited high diagnostic efficacy, surpassing the diagnostic efficacy of plasma p‐tau181 and NFL in all groups. This demonstrates that single‐cell immunomics facilitates the precise diagnosis of AD and DLB.

The differential features identified for each disease model represent the most significant characteristics of peripheral single‐cell immunity for that disease. The T cell subpopulation was the most distinct immune feature in the AD/HC group. The decline in T cells might be attributed to greater disease severity in AD patients and age‐related factors. The second differential feature was CD279 (PD1), whose expression was significantly higher in the T06 (CD4^+^ TEM) and T03 (CD4^+^ TCM) subpopulations compared with HC. Previous studies found that compared with wild‐type mice, the PD‐1 gene was one of the most significantly upregulated genes in microglia of amyloid precursor protein/presenilin‐1 (APP/PS1) mice, independent of age [35]. PD‐1 blockers have been shown to reduce brain Aβ plaque load and repeated anti‐PD‐1 treatment can have lasting beneficial effects on AD pathology [36]. PD‐1 is widely expressed in immune cells, with its signaling pathway being most prominent in activated T cells [37], consistent with our findings.

CD3 in the M03 subpopulation was the most significant differential feature in the DLB/HC model, with CD3 expression being significantly lower in DLB than HC in this subpopulation. However, CD3 is minimally expressed in myeloid cells. The minimal expression levels might be due to nonspecific staining, which causes confusion along with background noise within the detection range. The second differential immune feature was IKBα in the M01 (classical monocytes) subpopulation, whose expression was significantly lower in DLB than in HC. A study based on an α‐syn mouse model [38] showed that when microglia were treated with α‐syn, TLR2 recognized α‐syn and mediated a series of neuroinflammatory responses, upregulating MyD88, TRAF‐6, and TAK‐1, as well as IKBα and NF‐Kb, ultimately leading to apoptosis. α‐syn exhibits the highest TLR2 agonist activity, and TLR2 is primarily expressed by innate immune cells such as monocytes/macrophages and dendritic cells [39]. Based on limited theoretical grounds, we hypothesize that when α‐syn aggregates in DLB patients, TLR2 is highly expressed in the M01 subpopulation. However, downstream protein upregulation is obstructed due to the lack of microglial activation [40], resulting in the downregulation of IKBα expression.

IL‐17A in the NK02 subpopulation was the most significant differential feature in the AD/DLB model whose expression was significantly elevated in the AD group compared with the DLB group. A similar pattern was observed in the DLB/HC model, where it ranked as the third differential subpopulation, indicating the importance of this differential expression in the DLB population. Although IL‐17A is widely expressed in various neurodegenerative diseases [41], its expression in DLB has been rarely reported. In vitro experiments have confirmed that IL‐17A cannot exacerbate the loss of dopaminergic neurons without the involvement of activated microglia [42]. Silencing the IL‐17A receptor gene in microglia can achieve a similar result [43]. The lack of microglial activation in DLB brain tissue [40] is closely related to the low expression of IL‐17A. Additionally, HMGB1 has been detected in brain sections of DLB patients [44]. HMGB1 is typically used to mediate microglial activation, and the HMGB1A box can specifically bind α‐syn, inhibiting microglial activation and Th17 cell infiltration [45]. Our results showed that peripheral expression of IL‐17A in DLB patients was reduced, perhaps due to the lack or minimal activation of microglia in the brains of DLB patients. Additionally, the presence of high levels of the HMGB1A box or other substances in the CNS of DLB patients may inhibit microglial activation and steadily reduce IL‐17A levels. Previous studies found that peripheral IL‐17A levels were significantly elevated in both AD patients and transgenic AD model mice [46]. A peripheral blood study of AD patients [47] found that during the development of AD, early Th17 cells increased and IL‐17A secretion rose; however, IL‐17A levels decreased with disease progression and showed no difference compared with HC. Contrarily, IL‐17A levels were generally lower in DLB than in HC. Based on these theories, we believe that IL‐17A expression in the AD group is higher than that in the DLB group in the AD/DLB model, and our data showed that this difference was particularly significant in the NK02 subpopulation. CD8a was the secondary differential feature in the AD/DLB model, showing differences in the NK01 and NK03 subpopulations. In most subpopulations of the model, CD8a expression was lower in AD than in DLB. A study involving PD, DLB, AD, and HC populations [48] found that CD8a, detected through multiple proximity extension assay biomarkers in CSF, had strong diagnostic efficacy. Lower CD8a levels were associated with AD, while higher CD8a levels were associated with DLB. An immune model constructed with CD8a effectively distinguished these three diseases. The trend of CD8a expression levels and their relationship with disease in this study aligns with our findings and is reflected in the AD/DLB model.

At present, some studies have shown that there are some other methods for the differential diagnosis of AD and DLB. These methods mainly focus on brain imaging and CSF biomarkers. In brain imaging technology, ^18^F‐FDG PET is helpful in differentiating various types of neurodegenerative diseases [49]. The differential diagnosis of AD and DLB is made by observing the regions with reduced tracer uptake in ^18^F‐FDG PET imaging. AD shows reduced uptake in the parietal, temporal association cortices, posterior cingulate cortex, and precuneus, while DLB shows reduced uptake in the occipital lobe, especially in the primary visual cortex. Compared with single‐molecule immunodetection by cyTOF, ^18^F‐FDG PET, like other PET scans, such as Aβ PET, is limited by equipment and site restrictions and cannot be carried out on a large scale in the community. In addition, the imaging of Parkinson's disease dementia (PDD) in ^18^F‐FDG PET is similar to that of DLB, and the brain regions with reduced uptake in some DLB patients overlap with those in AD. These deficiencies will all reduce the effectiveness of ^18^F‐FDG PET in differential diagnosis.

In addition to ^18^F‐FDG PET, some other brain imaging techniques require larger sample support and more research to support in differentiating DLB from AD. For example, I‐2beta‐carbomethoxy‐3beta‐(4‐iodophenyl)‐N‐(3‐fluoropropyl) nortropane ((^123^)I‐FP‐CIT) has a relatively high accuracy in diagnosing DLB, especially in the moderate dementia stage, but its diagnostic value in the prodromal stage is relatively low, and an abnormal result cannot rule out the diagnosis of frontotemporal lobar dementia [50]. ^123^I‐Metaiodobenzylguanidine (MIBG) myocardial scintigraphy is used to evaluate sympathetic nerve damage, and it has good sensitivity and specificity in the dementia stage of DLB, but its diagnostic value in the prodromal stage is relatively low, and there is a lack of phase III studies for differentiating DLB from AD [51].

In terms of CSF biomarkers, in addition to the quantitative detection of Aβ and tau proteins mentioned above, there are currently some other CSF biomarkers used to distinguish AD from DLB. Neurosin is an α‐synuclein protease: its level is significantly lower in synucleinopathies (such as DLB, PD, PDD) than in AD patients and controls [52]. Markers related to neuroinflammation (YKL—40, IL—6): the level of YKL—40 in the CSF of AD patients is significantly higher than that in DLB patients and HC [53]; the level of IL—6 in the CSF of DLB patients is significantly lower than that in AD patients and non‐dementia controls [54]. Synaptic proteins (Chromogranin A (CgA), Neurogranin): CgA is significantly higher in DLB patients than in controls, PD and PDD patients [55], but further research is needed for comparison with AD patients; the level of neurogranin in the CSF of AD patients is significantly higher than that in DLB patients [53]. Although these CSF biomarkers are helpful for the differential diagnosis of AD and DLB, they are only small‐sample studies and require more data support and verification. Moreover, when distinguishing DLB, it is not easy to exclude synucleinopathies such as PD. Compared with single‐molecule immunodetection of peripheral blood by cyTOF, the collection of CSF is more difficult and risky, and the detection resolution also has no advantage.

Finally, our study shows that sex‐specific immune responses were observed in AD patients but were absent in DLB patients. A specific immune response in CD279 (PD1), particularly in the T06 (CD4^+^ TEM) subpopulation, was observed in male AD patients. Meanwhile, sex‐specific expressions in female AD patients were found in CD3 in the M01 and M05 subpopulations and CD27 in the M05 subpopulations. We believe that these sex‐specific immune responses may be related to the sex hormone‐specific effects on immune subpopulations or the expression of genes highly associated with AD risk in a sex‐specific manner. Studies have shown that CD4^+^ TEM can differentiate into Th1 cells, and androgens can significantly influence Th1 cell differentiation [56]. Postinfection levels of Th1‐related interferon‐gamma (IFNγ) are higher in male animals [57] and adult males [58] than in females, suggesting that androgens may participate in immune processes by regulating Th1 cell differentiation, thereby affecting CD4^+^ TEM. Another study [59] found a significant increase in the expression of AD‐related inflammatory response gene sets in monocytes from postmenopausal women. These highly expressed genes were correlated with the microglial gene expression profiles associated with Aβ plaques and phosphorylated tau protein load in AD brain tissues and significantly upregulated [60]. Our study found sex‐specific expressions in monocytes and basophils in female AD patients, which may be closely related to the susceptibility of female microglia to AD inflammatory genes and the high correlation of their monocytes with these specific gene expressions.

### 4.1 Limitations

This study has several limitations. First, the sample size of the subjects is relatively small and predominantly female. Second, this is a cross‐sectional study, lacking a longitudinal design, limiting the observation of changes in peripheral single‐molecule immune cells over time. Finally, the disease group lacked corresponding pathological biomarkers at enrollment. Our future studies will address these limitations by increasing the sample size, balancing the gender ratio, including pathological biomarkers for AD and DLB as enrollment criteria, and following up with all subjects, periodically testing their peripheral PBMCs. We will also include more research centers, incorporate studies of multiple cohorts, and compare and validate the results.

### 4.2 Conclusion

In summary, the current study applied CyTOF technology to analyze PBMCs of individuals with AD, DLB, and HC at a single‐cell resolution and found that AD was primarily characterized by a decrease in various T cell levels, with a total reduction in T cells being the most significant peripheral immune characteristics. Changes in peripheral subpopulations in DLB were not as pronounced as in AD, with only a partial decrease in T cell abundance and most subpopulations showing no significant difference compared with HCs. In terms of diagnosis, the peripheral single‐cell immune model demonstrated powerful diagnostic capabilities, surpassing those of peripheral plasma p‐tau181 and NFL. The lack of microglial activation in DLB brain tissue limits the expression of a series of peripheral immune cells. AD exhibits gender‐specific immune responses, with females and males expressing specific markers in certain subpopulations. The peripheral monomolecular immune characteristics of neurodegenerative diseases such as AD and DLB showed considerable differences compared with those from previous immunological research, aiding in the precise clinical diagnosis and uncovering more disease immune characteristics. Nonetheless, these findings should be validated prospectively in further research.

## Author Contributions

S.H. and W.C. conceived the project and designed the experiments. D.Z. and C.Q. conducted the CyTOF and ELISA experiments and collected and organized the experimental data. C.Q., M.W., and X.M. participated in data analysis. G.P., D.Z., and C.Q. were involved in the inclusion and collection of peripheral blood samples. C.Q., H.Y., W.Y., G.P., W.C., and S.H. wrote the manuscript with suggestions and feedback from all authors. S.H., as the guarantor, is responsible for the overall content.

## Conflicts of Interest

The authors declare no conflicts of interest.

## Supporting information


**Figure S1.** Baseline characteristics of AD, DLB patients, and HC.
**Figure S2.** Comparison of the most significant immune features among the AD/HC, DLB/HC, and AD/DLB group models.
**Table S1.** panel information.
**Table S2.** Immune cell subpopulation information.
**Table S3.** Normality test of clinical data.

## Data Availability

The data that support the findings of this study are available from the corresponding author upon reasonable request.
