# Supplementary material for: Peripheral Single‐Cell Immune Characteristics Contribute to the Diagnosis of Alzheimer's Disease and Dementia With Lewy Bodies
Source: CNS Neurosci Ther. 2025 Jan 3;31(1):e70204. doi: 10.1111/cns.70204 (PMC11702477; doi:10.1111/cns.70204)
Supplement: Supplementary file 1 — Figure S1. Baseline characteristics of AD, DLB patients, and HC. Figure S2. Comparison of the most significant immune features among the AD/HC, DLB/HC, and AD/DLB group models. Table S1. panel information. Table S2. Immune cell subpopulation information. Table S3. Normality test of clinical data. [file CNS-31-e70204-s001.pdf]

Supplementary Materials

Supplementary Figures

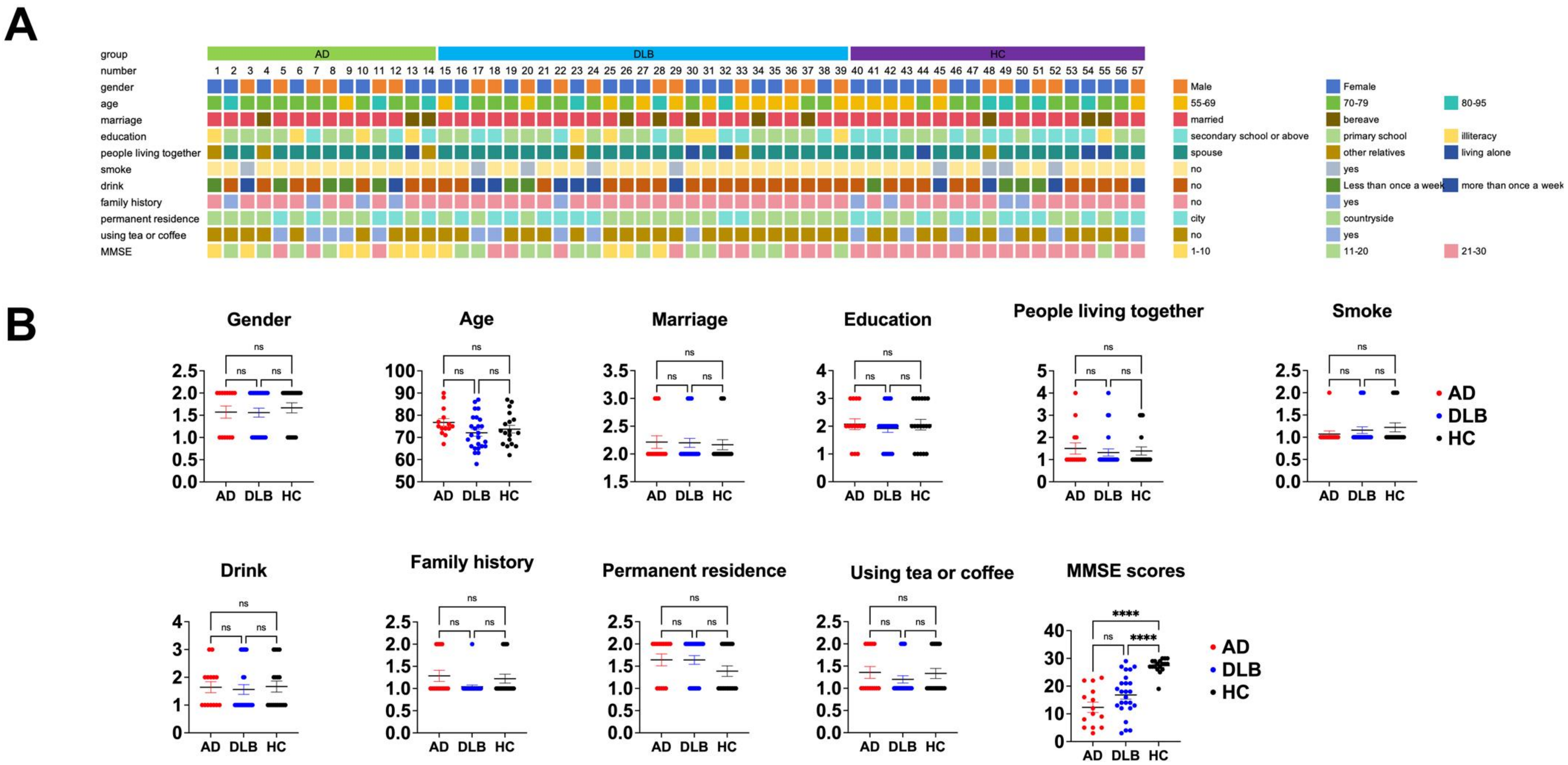

Figure S1. Baseline characteristics of AD, DLB patients, and HC. (A) A color-coded block represents the gender, age, marital status, education, residence, smoking, drinking, family history, place of long-term residence, tea/coffee consumption, and MMSE scores of 57 subjects across the three groups, with annotations for each color block on the right. **(B)** Comparison of 11 baseline characteristics among three groups. One-Way ANOVA analysis was used for age. The Kruskal-Wallis test was applied for MMSE scores. For pairwise comparisons, the independent-samples t-test or Mann-Whitney U test was adopted according to the normality. And other characteristics were evaluated using the chi-square test, as shown in the dot plots. **(B)** ****p<0.0001

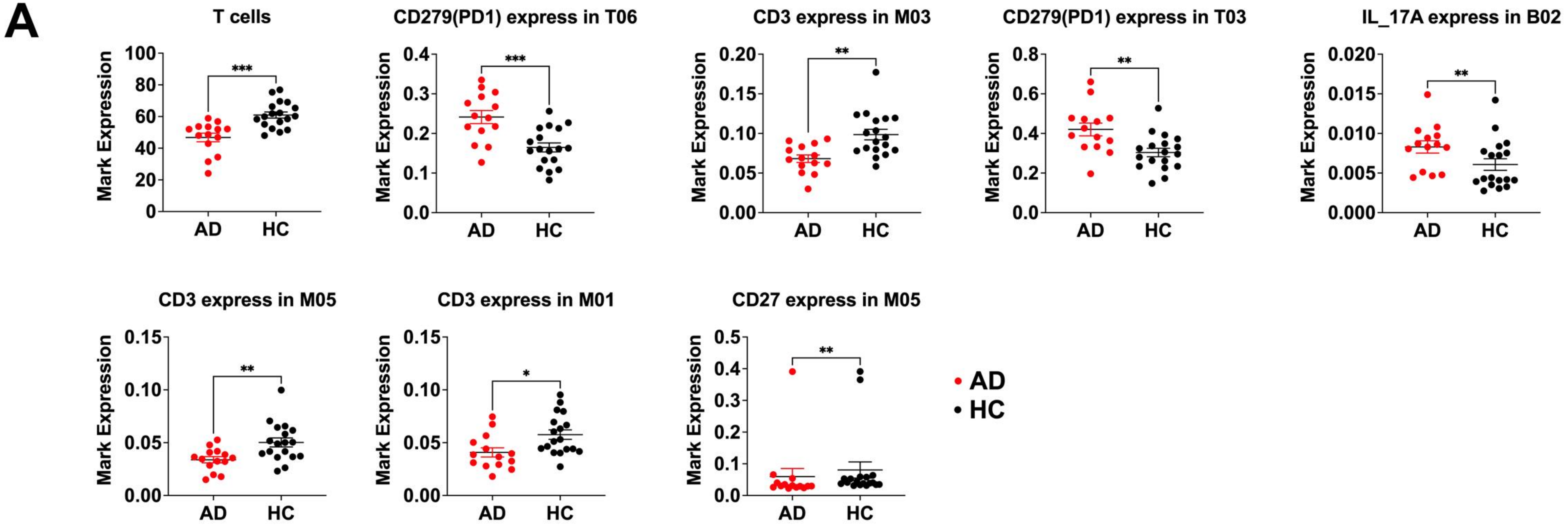

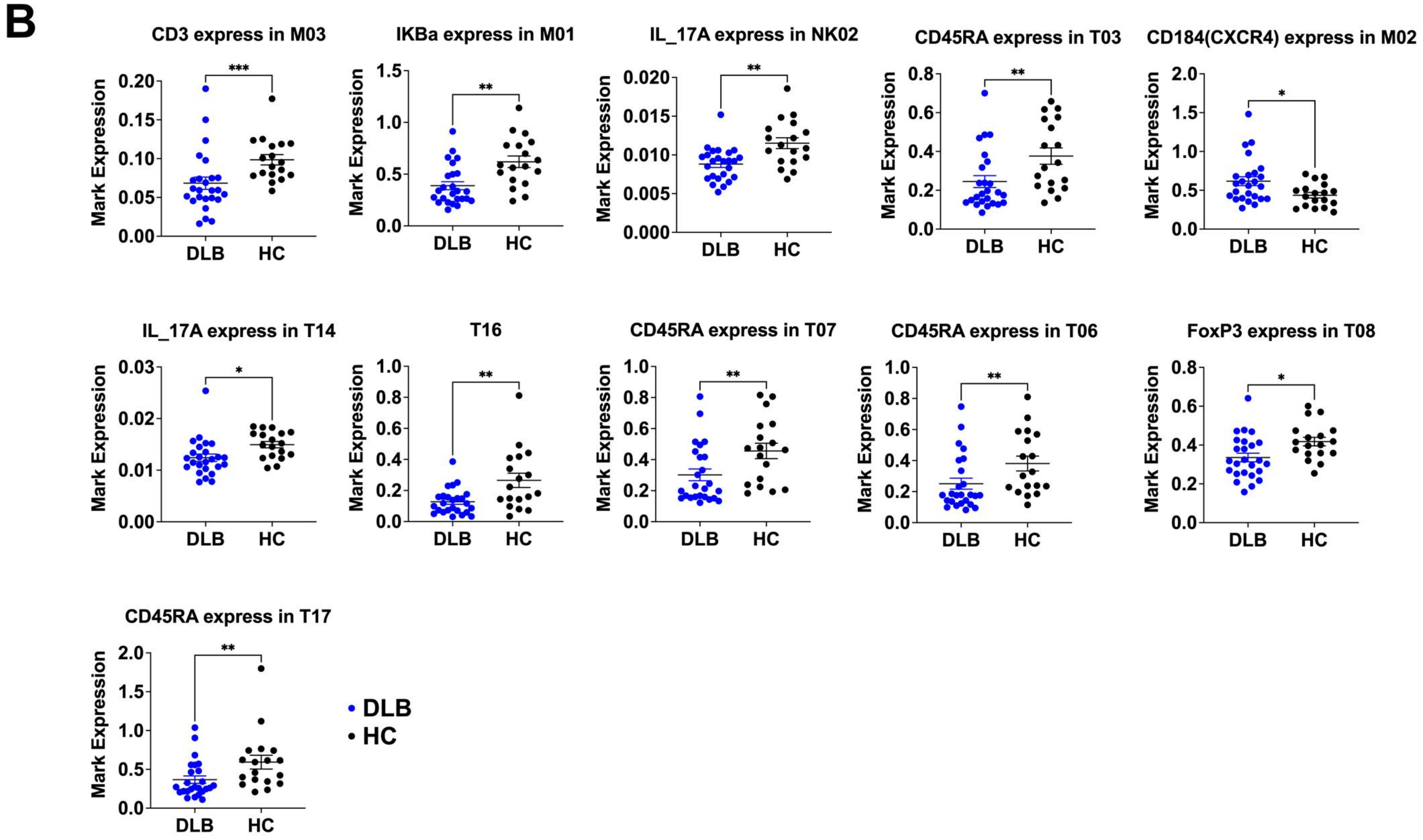
B
CD3 express in M03
IKBa express in M01
IL_17A express in NK02
CD45RA express in T03
CD184(CXCR4) express in M02
IL_17A express in T14
T16
CD45RA express in T07
CD45RA express in T06
FoxP3 express in T08
CD45RA express in T17
Mark Expression
DLB
HC
***
**
*

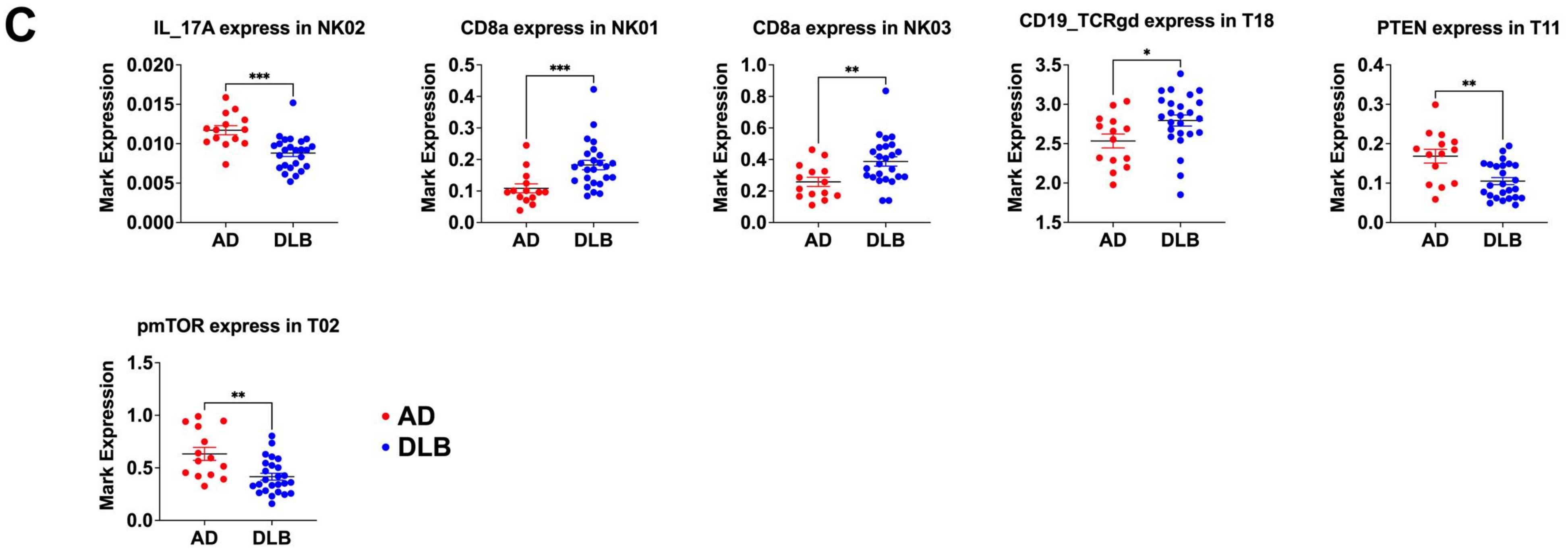

Figure S2. Comparison of the most significant immune features among the AD/HC, DLB/HC, and AD/DLB group models. (A-C) Comparison of the expression values of the most significantly differential markers and clusters within the iEN models for the AD/HC group **(A)**, the DLB/HC group **(B)**, and the AD/DLB group **(C)**. For the two groups of data that are normally distributed, an independent-samples t-test shall be used. For the case where one or both groups of data are not normally distributed, the Mann-Whitney U test shall be adopted. Calculate the P value and mark it as *p < 0.05, **p < 0.01, ***p < 0.001.

Supplementary Tables

Table.S1

Serial Number	marker	Metal channel	clone	Vendor	Cat	Staining
1	CD45	89Y	HI30	BioLegend	304002	surface
2	CD3	115In	UCHT1	BioXcell	BE0231	surface
3	CD56	141Pr	NCAM16.2	BD	559043	surface
4	TCR γ/δ	142Nd	5A6.E9	Homemade	NA	surface
5	CD19	142Nd	HIB19	BioLegend	333802	surface
6	CD27	143Nd	O323	BioLegend	302802	surface
7	pPLCγ2	144Nd	K86-689.37	Fluidigm	3144015A	Intracellular
8	pIKB alpha	145Nd	EPR6235(2)	Abcam	ab240059	Intracellular
9	BCL-2	146Nd	100	Thermo	138800	Intracellular
10	pSTAT5	147Sm	47	Fluidigm	3147012A	Intracellular
11	TAK1	148Nd	28H25L68	Thermo	700113	Intracellular
12	CD25	149Sm	24212	RD	MAB1020	surface
13	CD14	150Nd	M5E2	BioLegend	301810	surface
14	CD38	151Eu	HIT2	BioLegend	303502	surface
15	pAkt	152Sm	D9E	Fluidigm	3152005A	Intracellular
16	pSTAT1	153Eu	4a	Fluidigm	3153005A	Intracellular
17	CCR7	154Sm	G043H7	BioLegend	353222	surface
18	CD45RA	155Gd	HI100	BioLegend	304102	surface
19	CD184	156Gd	12G5	BioLegend	306502	surface
20	IL-4	157Gd	MP4-25D2	BioLegend	500829	Intracellular

21	pSTAT3	158Gd	4/p-Stat3	Fluidigm	3158005A	Intracellular
22	PTEN	159Tb	217702	R&D	MAB847	Intracellular
23	pmTOR	160Gd	EPR427(N)	Abcam	ab232486	Intracellular
24	MyD88	161Dy	4D6	Thermo	MA5-16231	Intracellular
25	FoxP3	162Dy	PCH101	eBioscience	14-4776-82	Intracellular
26	TRAF-6	163Dy	326019	R&D	MAB3284-100	Intracellular
27	IKB alpha	164Dy	L35A5	Fluidigm	3164004A	Intracellular
28	IFN-γ	165Ho	B27	BioLegend	506521	Intracellular
29	pNFkBp65	166Er	K10-895.12.50	Fluidigm	3166006A	Intracellular
30	pTAK1	167Er	EPR2863	Abcam	ab239974	Intracellular
31	CD11c	168Er	BU15	BioLegend	337202	surface
32	CD49d	169Tm	9F10	BioLegend	304302	surface
33	CD127	170Er	A019D5	BioLegend	351302	surface
34	PD-1	171Yb	EH12.2H7	BioLegend	329926	surface
35	AIFM1	172Yb	7F7AB10	Invitrogen	45-6200	Intracellular
36	IL-17A	173Yb	BL168	BioLegend	512331	Intracellular
37	DJ-1	174Yb	7	Invitrogen	MA5-29462	Intracellular
38	CD16	175Lu	3G8	BioLegend	302014	surface
39	HLA-DR	176Yb	L243	BioLegend	307612	surface
40	CD4	197Au	RPA-T4	BioLegend	300516	surface
41	CD8a	198Pt	RPA-T8	BioLegend	301018	surface
42	CD11b	209Bi	M1/70	BioLegend	301214	surface

Table.S1.panel information.42 panel information include marker names, Metal channel, clone, Vendor, Cat and Staining (CD19 and TCRγ/δ are the same channel)

Table.S2

Subgroup number	Subgroup name	Cellular immune typing
T01	CD4+T Navie	CD3+CD4+CD27+CD25-CD45RA+
T02	CD4+TCM	CD3+CD4+CD27+CD25-CD45RA-CD184mod
T03	CD4+TCM	CD3+CD4+CD27+CD25-CD45RA-CD184dim
T04	CD4+TEM	CD3+CD4+CD27-CD25-CD45RA-CD127dimCD56+
T05	CD4+TEM	CD3+CD4+CD27-CD25-CD45RA-CD127dimCD56-
T06	CD4+TEM	CD3+CD4+CD27-CD25-CD45RA-CD127bright
T07	Treg	CD3+CD4+CD27+CD25+
T08	CD8+T Navie	CD3+CD8+CD27+CCR7+
T09	CD8+TEMRA	CD3+CD8+CD27-CCR7-CD56-CD11b-
T10	CD8+TEMRA	CD3+CD8+CD27-CCR7-CD56+
T11	CD8+TEMRA	CD3+CD8+CD27-CCR7-CD56-CD11bbright
T12	CD8+TCM	CD3+CD8+CD27-CCR7+
T13	CD8+TEM	CD3+CD8+CD27+CCR7-CD127-
T14	CD8+TEM	CD3+CD8+CD27+CCR7-CD127mod
T15	CD8+TEM	CD3+CD8+CD27+CCR7-CD127bright
T16	DNT	CD3+CD4-CD8-CD38+
T17	DPT	CD3+CD4+CD8+
T18	γδT	CD3+CD4-CD8-CD38-
B01	memory	CD3-CD19+CD27+
B02	naive	CD3-CD19+CD27-
NK01	NK cells	CD3-CD19-CD56+CD11c-CD197+
NK02	NK cells	CD3-CD19-CD56+CD11c-

NK03	NK cells	CD3-CD19-CD56+CD11c+CD45RAbright
NK04	NK cells	CD3-CD19-CD56+CD11c+CD45RAmod
M01	classical Monocytes	CD3-CD19-CD14+CD16-
M02	non-classical Monocytes	CD3-CD19-CD14+CD16+
M03	cDC	CD3-CD19-HLA_DR+CD11c+
M04	pDC	CD3-CD19-HLA_DR+CD11c-
M05	Basophils	CD3-CD19-HLA_DR-

Table.S2.Immune cell subpopulation information. Immune cell subpopulation information include Subgroup number，Subgroup name and Cellular immune typing。

Table.S3

Normality Test				
		K-S（P value）		
Figure	Group	AD	DLB	HC
F1C	T	0.076	0.135	0.200
	B	0.200	0.200	0.200
	NK	0.200	0.200	0.200
	Myeloid	0.200	0.200	0.200
F2D	CD4+T	0.200	0.159	0.200
	CD8+T	0.200	0.016	0.200
	DNT	0.193	0.200	0.200
	DPT	0.015	<.001	<.001
	gdT	0.080	0.116	0.011
F2E	CD4+T Navie	0.018	0.093	0.200
	CD4+TCM	0.200	0.200	0.200

	CD4+TEM	0.200	0.200	<.001
	Treg	0.113	0.072	0.200
	CD8+T Navie	0.004	0.043	0.054
	CD8+TEMRA	0.200	<.001	0.117
	CD8+TCM	0.007	0.002	0.015
	CD8+TEM	0.009	0.200	0.200
F2F	T01	0.018	0.093	0.200
	T02	0.107	0.144	0.004
	T03	0.200	0.200	0.200
	T04	0.031	<.001	<.001
	T05	0.036	0.200	0.012
	T06	0.011	0.014	0.200
	T07	0.113	0.072	0.200
	T08	0.004	0.043	0.054
	T09	0.004	0.001	0.003
	T10	0.029	0.001	0.030
	T11	0.200	0.200	0.192
	T12	0.007	0.002	0.015
	T13	0.059	0.169	0.200
	T14	0.063	0.200	0.154
	T15	0.200	<.001	0.002
	T16	0.193	0.200	0.200
	T17	0.015	<.001	<.001

	T18	0.080	0.116	0.011
F3C	B01	<.001	0.170	0.200
	B02	0.065	0.200	0.200
F3G	NK01	0.135	0.007	0.021
	NK02	0.200	0.058	0.200
	NK03	0.200	<.001	0.003
	NK04	<.001	0.002	<.001
F3K	M01	0.200	0.200	0.200
	M02	0.176	0.179	0.200
	M03	0.002	0.022	0.200
	M04	0.200	0.033	0.130
	M05	0.200	0.009	0.008
S1B	Age	0.131	0.200	0.200
	MMSE	0.200	0.200	0.025
S2A	T	0.076	N/A	0.200
	T06CD279(PD1)	0.200	N/A	0.200
	M03CD3	0.200	N/A	0.200
	T03CD279(PD1)	0.200	N/A	0.200
	B02IL_17A	0.200	N/A	0.002
	M05CD3	0.200	N/A	0.200
	M01CD3	0.200	N/A	0.200
	M05CD27	<.001	N/A	<.001
S2B	M03CD3	N/A	0.001	0.200

	M01IKBa	N/A	0.012	0.200
	NK02IL_17A	N/A	0.200	0.200
	M05CD38	N/A	0.200	0.200
	T03CD45RA	N/A	<.001	0.200
	M02CD184.CXCR4.	N/A	0.114	0.200
	T14IL_17A	N/A	0.071	0.200
	T16	N/A	0.200	0.200
	T07CD45RA	N/A	0.004	0.200
	T06CD45RA	N/A	<.001	0.048
	T08FoxP3	N/A	0.200	0.200
	T17CD45RA	N/A	0.002	0.028
S2C	NK02IL_17A	0.200	0.200	N/A
	NK01CD8a	0.007	0.183	N/A
	NK03CD8a	0.200	0.200	N/A
	T18CD19_TCRgd	0.200	0.200	N/A
	T11PTEN	0.200	0.114	N/A
	T02pmTOR	0.200	0.156	N/A

Table.S3.Normality test of clinical data. Perform the Kolmogorov-Smirnov test on all clinical data and record the p-values in the table.
